# Parental education and willingness to vaccinate children with self-paid vaccines in Hangzhou, China: a cross-sectional study

**DOI:** 10.3389/fpubh.2026.1804018

**Published:** 2026-10-14

**Authors:** Linbo Xie, Zhihan Wu, Qiao Zhang, Kehan Wang, Ziyan Zhu, Zihan Wang, Peiyao Li, Siyu Chen, Junfang Xu, Min Yuan, Jiming Zhu

**Affiliations:** 1 Institute of Basic Research in Clinical Medicine, China Academy of Chinese Medical Sciences, Beijing, China; 2 Vanke School of Public Health, Tsinghua University, Beijing, China; 3 Institute for Healthy China, Tsinghua University, Beijing, China; 4 School of Public Health, Capital Medical University, Beijing, BM, China; 5 Department of Medicine, Rutgers University, New Brunswick, NJ, United States; 6 Nanyang Technological University, Singapore; 7 School of Pharmaceutical Sciences, Tsinghua University, Beijing, China; 8 Department of Economic Management, Huazhong Agricultural University, Wuhan, China; 9 School of Public Health, Zhejiang University School of Medicine, Hangzhou, China; 10 Department of Health Data Science, Anhui Medical University, Hefei, Anhui, China; 11 MOE Key Laboratory of Population Health Across Life Cycle, Hefei, Anhui, China; 12 China Medical Board, Beijing Office, Beijing, China

**Keywords:** children, family income, mediation analysis, self-paid vaccines, vaccine hesitancy

## Abstract

**Background:**

Childhood vaccination is a cost-effective intervention for preventing vaccine-preventable diseases. However, the specific mechanisms by which parental education level influences willingness to vaccinate (WTV) for self-paid child vaccines remain unexplored.

**Objectives:**

This study investigates the mediating roles of family income, vaccine awareness, and trust in self-paid child vaccines in the relationship between parental education and WTV for self-paid child vaccines, including pentavalent, EV71, varicella, and flu vaccines in China.

**Methods:**

A survey of 2,212 households with children under five in Hangzhou, Zhejiang Province, was conducted. Mediation analysis was used to assess the effects of family income, vaccine awareness, and trust on WTV.

**Results:**

Our findings indicate that family income was an important pathway linking parental education to WTV, particularly for flu and varicella vaccines. After family income was introduced, the association between parental education and WTV was attenuated, suggesting that income may play an important mediating role. For the pentavalent vaccine, vaccine awareness and trust in self-paid vaccines also attenuated this association. These findings suggest that, although economic factors are important, non-economic factors such as vaccine awareness and trust in self-paid vaccines should also be considered.

**Conclusion:**

Addressing financial barriers is crucial for increasing vaccine uptake for self-paid child vaccines in developing countries. Public health strategies should prioritize financial assistance programs and targeted communication efforts to build trust and ensure equitable access to essential vaccinations.

## 1 Introduction

Vaccination stands as a pivotal investment in public health, offering both individual protection and community-wide benefits by preventing disease and enhancing public health (1). A recent modeling study estimated that vaccination has averted approximately 154 million deaths globally since 1974, including 146 million deaths among children younger than 5 years (2). Childhood vaccination is therefore closely linked to global efforts to reduce under-5 mortality, including the Sustainable Development Goal target of reducing under-5 mortality to 25 or fewer deaths per 1,000 live births by 2030 (3). In many high-income countries (HICs), publicly funded immunization programs have reduced direct financial barriers to routine childhood vaccination. The United States Vaccines for Children program provides recommended vaccines free of charge to eligible children, while Canada and Germany have also implemented broad publicly funded or nationally recommended childhood immunization programs (4–6). However, the experience of high-income countries (HICs) also shows that financial coverage alone does not guarantee optimal vaccine uptake. Vaccine hesitancy remains an important barrier in these settings and may be related to confidence, complacency, convenience, knowledge gaps, and concerns about vaccine safety or effectiveness (7).

In contrast, evidence from low- and middle-income countries (LMICs) suggests that under-vaccination is often shaped by a broader combination of health-system, access, financing, knowledge-related, and confidence-related barriers. Studies from Uganda and Zambia have shown that low vaccine coverage or uptake may be linked to access barriers, inadequate funding, vaccine supply constraints, limited community education, and insufficient vaccine confidence (8, 9). Vaccination may also help reduce antimicrobial resistance by preventing infections and decreasing unnecessary antibiotic use (10). These findings suggest that, compared with high-income countries, vaccine willingness and uptake in LMICs settings may be more strongly influenced by both information- and affordability-related barriers.

China provides an important middle-income country context for examining willingness to use self-paid childhood vaccines. China’s childhood vaccination system distinguishes between National Immunization Program (NIP) vaccines and non-NIP vaccines. NIP vaccines are mandatory and provided free of charge to eligible children, whereas non-NIP vaccines are usually voluntary and self-funded (11). According to China’s current childhood immunization schedule, NIP vaccines include hepatitis B vaccine, Bacille Calmette–Guérin vaccine, poliovirus vaccines, diphtheria-tetanus-pertussis/diphtheria-tetanus vaccines, measles-mumps-rubella vaccine, Japanese encephalitis vaccines, meningococcal polysaccharide vaccines, and hepatitis A vaccines (12). Notably, the measles-mumps-rubella vaccine is included in the NIP schedule, with two routine doses recommended at 8 and 18 months of age (12).

Although China’s NIP has achieved high coverage for many routine childhood vaccines, several important childhood vaccines remain outside the NIP and are mainly paid for self-paid by families (12). These non-NIP vaccines include pneumococcal conjugate vaccine, rotavirus vaccine, *Haemophilus influenzae* type b vaccine, and varicella vaccine, all of which are available in China but not yet universally included in the publicly funded program (12). Previous studies have reported inequalities in non-NIP vaccine coverage in China, suggesting that family socioeconomic status (SES) and affordability may influence uptake (13). Therefore, in China, low uptake of some self-paid vaccines may reflect not only mistrust or insufficient knowledge, but also families’ financial ability to afford vaccination (14).

Vaccine willingness depends on factors like information (15, 16), education level (17), region (18), economic considerations (19), and so on. Among these, education level has been consistently identified as a key predictor of vaccine acceptance in many studies. In Sweden, parents with a lower education level are more likely to be unsure about vaccinating their kids (16). In Houston, TX, USA, women who are going to have a baby and have a lower education level are more hesitant about vaccines (20). In China, parents with higher education levels are less hesitant about vaccines (21).

Previous studies have provided frameworks for understanding the mediating effect of vaccine willingness. One study developed a mediation framework to examine how behavioral and cognitive attitudes influence influenza self-vaccination in Poland (22). Similarly, in China, a study conducted a moderated mediation model analysis to explore how attitudes, subjective norms, and perceived behavioral control mediate vaccination intention and behavior among the general public (23). Also, a recent study conducted in China on mediation analysis between SES and vaccine hesitancy indicated the importance of perceptions of social support and health literacy (24). However, the mediation pathway by which education influences vaccine willingness has not been clarified.

We explore whether family income is one possible route from parental education to willingness to take a self-paid vaccine in China. In addition to vaccine information and trust, household financial resources may also be helpful for understanding socioeconomic differences in vaccine willingness (25, 26).

In this study, we used mediation analysis as an exploratory analytical approach to examine whether family income, vaccine-related information, and trust may serve as potential pathways linking parental education with willingness to use self-paid vaccines. Instead of finding completely new factors, we wanted to go beyond single-factor associations and provide context-level evidence that this might also contribute to vaccine willingness in China. We also considered heterogeneity across different self-paid vaccines, since the information, trust, and financial resources that a household may have are related to the safety, efficacy, and familiarity of each vaccine (27). Given the observational nature of the data, these pathway estimates should be interpreted cautiously and as hypothesis-generating rather than causal.

## 2 Method

### 2.1 Survey and dataset

Between 22 March 2023 and 30 June 2023, we conducted a cross-sectional, face-to-face survey among 2,212 households in Hangzhou city, Zhejiang Province, China. Hangzhou was selected as the study setting because the survey was conducted within an established fieldwork network for childhood vaccination services, and local vaccination clinics provide both compulsory and self-paid vaccines. This setting allowed us to recruit parents or caregivers attending routine childhood vaccination services and to examine willingness to vaccinate children with selected self-paid vaccines in a real-world service context.

To improve the sample’s diversity in Hangzhou, three districts/counties were selected based on different levels of economic development. Vaccination sites, including community health centers and maternity and childcare hospitals, were then selected within these districts for data collection. These vaccination centers provide both compulsory and self-paid childhood vaccines; therefore, respondents included parents or caregivers accompanying their children for either compulsory or self-paid vaccination services. This sampling strategy may have reduced, although not eliminated, the possibility of recruiting only parents with a strong preference for self-paid vaccination.

Parents or caregivers who accompanied children to the vaccination center were invited to participate in the survey. Observations were excluded if no parent or eligible caregiver was present with the child, or if the questionnaire contained incomplete or illogical responses. Data were collected using a paper-based, structured questionnaire comprising four sections: self-paid vaccination-related intentions and reasons; vaccine hesitancy; sociodemographic characteristics of caregivers and children and willingness to vaccinate children with selected self-paid vaccines.

Given the observational and cross-sectional nature of the data, mediation analysis was used to explore potential indirect pathways and was interpreted cautiously rather than as evidence of causal mediation.

### 2.2 Measures

The outcome variables are parents’ willingness for their children to be vaccinated by the pentavalent vaccine, Enterovirus 71 (EV71) vaccine, varicella vaccine, and flu vaccine, respectively. For example, for the pentavalent vaccine, the survey question asks: given the current self-paid price, what is your willingness for your children to be vaccinated (rated 0–10, with 10 being the highest willingness). The main explanatory variable is a dummy variable, indicating whether any of the parents in this family has a university bachelor’s degree. These four vaccines were selected before data collection because they were commonly available self-paid childhood vaccines at local vaccination clinics during the survey period and applicable to children in the target age range. They were also chosen to cover several common self-paid vaccination scenarios, including combination vaccination, prevention of hand, foot, and mouth disease, prevention of varicella, and seasonal influenza vaccination. This allowed us to examine whether parental willingness differed across different types of self-paid childhood vaccines.

We propose three potential mechanisms. The first potential mechanism was household income. Monthly household income was collected using five pre-specified ordered categories in the questionnaire: <5,000 Renminbi (RMB), 5,000–10,000 RMB, 10,000–20,000 RMB, 20,000–30,000 RMB, and >30,000 RMB. These categories were defined before data collection and were not generated based on the observed income distribution in the study sample. Categorical income reporting was used because household income is a sensitive item in face-to-face surveys, and broad response categories may reduce respondent burden and improve response completeness. The income categories were used as approximate indicators of household economic resources, while also helping maintain a sufficient sample size within each category for regression and exploratory mediation analyses. The second mechanism suggests that having a university degree may enhance access to information and awareness about self-paid vaccines. We measure this through whether respondents heard of the proposed vaccines. Additionally, for the pentavalent vaccine, we specifically ask if they understand the difference between pentavalent and single-valent vaccines. The third mechanism proposes that a university degree may increase trust in self-paid vaccines, thereby boosting willingness to vaccinate. We capture trust levels using dummy variables derived from responses to questions such as “Do you think self-paid vaccines are essential for your child’s health?” “Are self-paid vaccines effective?” “Do you believe self-paid vaccines are important for others in society?” and “Are you concerned about the side effects of self-paid vaccines?” Furthermore, for the pentavalent vaccine, we directly ask, “Do you trust the pentavalent vaccine?”

Our analysis incorporates several control variables: the number of children in the family, whether the family received doctors’ recommendations for self-paid vaccines, the ages of the father and mother, and their travel costs associated with getting the vaccines. Additionally, we include fixed effects for the family’s religious beliefs, the father’s occupation, and the mother’s occupation.

### 2.3 Questionnaire development

Data were collected using a paper-based structured questionnaire in Chinese. The questionnaire was developed based on the study objectives, previous literature on parental vaccine hesitancy and self-paid childhood vaccination, and a previously reported questionnaire from the same survey program. Researchers with expertise in public health, vaccination, and reviewed the questionnaire items. The vaccine hesitancy section was adapted from the Vaccine Hesitancy Scale, whereas the willingness-to-vaccinate and willingness-to-pay items were designed to align with the specific research aims of the present study and the local vaccination service context. Before the main analysis, the initial paper-based responses were used to assess item clarity, response feasibility, logical consistency, and internal consistency (28).

### 2.4 Regression analysis

We utilize Ordinary Least Squares (OLS) regressions to empirically investigate whether the three proposed mechanisms are valid, following these steps: Initial Test: Firstly, we aim to establish whether possessing a university degree potentially enhances parents’ willingness to vaccinate their children with self-paid vaccines. Inclusion of Mediators: Next, we incorporate the mediator variables linked to the proposed mechanisms to assess whether the coefficient estimates for “university” experience are significant reduced. Impact on Mediators: Finally, we examine the potential influence of a university degree on these mediator variables. Detailed descriptions of the regression functions are available in Supplementary material S1. We use Stata (version 17 MP) to conduct the analysis.

## 3 Results

### 3.1 Summary statistics

Table 1 presents the summary statistics. The average age of fathers in these families is 34.59 years, while the average age of mothers is 33.09 years. On average, families have 1.5 children. There is a relatively low willingness among parents for their children to receive pentavalent vaccines (4.83 ± 3.71) compared with EV71 vaccines (6.18 ± 3.66), varicella vaccines (6.83 ± 3.51), and flu vaccines (6.60 ± 3.51). Despite this, pentavalent vaccines are the most recognized (0.72 ± 0.45) among the four self-paid vaccines, though only about half of the respondents understand the difference between pentavalent and singular vaccines. Approximately 72% of families trust pentavalent vaccines. Overall, 60% of families have at least one parent with a university bachelor’s degree. Regarding income levels, the range of “10,001–20,000” includes the largest number of observations.

**Table 1:** Descriptive statistics of sample characteristics and parental perceptions of self-paid vaccines.

Variables	*N* (%)	Mean	SD	Minimum	Maximum
Outcome variables					
WTV of the pentavalent vaccine	2,081	4.831	3.710	0	10
WTV of EV71 vaccine	2,084	6.179	3.658	0	10
WTV of the varicella vaccine	2,083	6.832	3.513	0	10
WTV of flu vaccine	2,083	6.598	3.608	0	10
Explanatory variable					
University degree	2,069	0.600	0.490	0	1
Mediator variable: family income					
Family income level (RMB)	2,074				
Less than 5,000	94 (4.53)				
5,000 to 10,000	498 (24.01)				
10,001 to 20,000	758 (36.55)				
20,001 to 30,000	371 (17.89)				
Greater than 30,000	353 (17.02)				
Mediator variables: information and awareness					
Heard about the pentavalent vaccine	2,101	0.722	0.448	0	1
Heard about the EV71 vaccine	2,101	0.165	0.371	0	1
Heard about the varicella vaccine	2,101	0.638	0.481	0	1
Heard about the flu vaccine	2,101	0.672	0.470	0	1
Know the difference between penta- and singular vaccines	2,003	0.497	0.500	0	1
Mediator variables: trust in self-paid vaccines					
Vital for children’s health	2,082	0.718	0.450	0	1
Trust the effectiveness	2,082	0.735	0.441	0	1
Vital for the public	2,081	0.646	0.478	0	1
Worry about side effects	2,073	0.475	0.500	0	1
Prefer imported vaccines	2,085	0.447	0.497	0	1
Trust the pentavalent vaccine	2,103	0.718	0.450	0	1
Control variables					
Number of children	2,098	1.528	0.571	1	7
Family member is a medic	2,085	0.148	0.355	0	1
Doctor recommended	2,075	0.315	0.465	0	1
Father’s age (by 2023)	2,074	34.593	5.399	21	60
Mother’s age (by 2023)	2,065	33.091	4.882	19	61
Travel cost (RMB)	2,097				
Less than 10	902 (43.01)				
10–20	802 (38.25)				
20–30	265 (12.64)				
30–40	70 (3.34)				
Greater than 40	58 (2.77)				

SD, standard deviation.

We also have information on parental occupations and the religions of family members, which will serve as fixed effects in the regression analysis.

### 3.2 Regression analysis results

Table 2 illustrates the impact of holding a university degree on the willingness to vaccinate with pentavalent vaccines. The initial analysis in the first column serves as the benchmark regression, revealing a positive association between having a university degree and vaccination willingness. To explore potential mediators, we then introduce family income as an additional variable. As shown in column (2) of Table 2, the inclusion of income leads to a decrease in the university degree coefficient from 0.914 to 0.613, suggesting that income may be a mediating pathway. Again, building on the benchmark regression (1), we incorporate variables related to information and awareness in column (3), and the coefficient for university degree decreases to 0.536. Similarly, in column (4), we include variables that measure trust, which results in the university degree coefficient decreasing to 0.528. When we include all variables capturing these three mechanisms—income, information, and trust—as shown in column (5), the coefficient of the university degree becomes insignificant. This indicates that the combined effect of these pathways fully accounts for the influence of education on vaccination willingness.

**Table 2:** Parental education and willingness to vaccinate with pentavalent vaccines.

Variables	Dependent variable: willingness to be vaccinated against pentavalent vaccination				
	Model 1	Model 2	Model 3	Model 4	Model 5
University degree	0.914*** (0.202)	0.0613*** (0.208)	0.536*** (0.203)	0.528*** (0.189)	0.212 (0.199)
Family income		0.483*** (0.082)			0.378*** (0.079)
Heard about it			0.964*** (0.206)		0.501** (0.202)
Know the difference			1.502*** (0.183)		0.625*** (0.193)
Vital for children				0.496** (0.236)	0.462*** (0.240)
Effective				0.921*** (0.228)	0.932*** (0.234)
Vital for the public				0.361* (0.201)	0.316 (0.207)
Worry about side effects				−0.432*** (0.155)	0.400** (0.079)
Prefer imported				1.058*** (0.166)	0.848*** (0.171)
Trust				1.795*** (0.816)	1.405*** (0.206)
Observations				1,957	1,860
Adjusted *R*^2^				0.197	0.220

*p < 0.01, **p < 0.05, ***p < 0.01. Heteroskedasticity-robust standard errors are provided in parentheses. All models include the number of children, religious belief, whether any family member is a medic, doctor’s recommendation on self-paid vaccines, travel cost, father’s occupation, mother’s occupation, father’s age, and mother’s age.

Table 3 presents the mediating analysis for EV71 vaccines. Column (1) of Table 3 indicates a positive association between possessing a university degree and parents’ willingness to vaccinate their children with EV71 vaccines. When the income variable is introduced in column (2), the coefficient estimate for the university degree becomes insignificant, suggesting that income is a primary pathway mediating this relationship. This finding is further supported by columns (3) and (4), which show that adding variables related to information and trust levels nearly does not alter the coefficient estimate for the university degree.

**Table 3:** Parental education and willingness to vaccinate with EV71 vaccines results of regression analysis.

Variables	Dependent variable: willingness to be vaccinated of varicella vaccination				
	Model 1	Model 2	Model 3	Model 4	Model 5
University degree	0.608*** (0.202)	0.307 (0.208)	0.597*** (0.202)	0.525*** (0.198)	0.231 (0.202)
Family income		0.486*** (0.081)			0.451*** (0.079)
Heard about it			0.323 (0.222)		0.228 (0.218)
Vital for children				0.884*** (0.244)	0.852*** (0.243)
Effective				1.107*** (0.247)	1.116*** (0.246)
Vital for the public				0.348* (0.206)	0.342* (0.207)
Worry about side effects				−0.479*** (0.161)	−0.471*** (0.160)
Observations	1,995	1,980	1,994	1,971	1,955
Adjusted *R*^2^	0.048	0.064	0.048	0.103	0.118

*p < 0.01, **p < 0.05, ***p < 0.01. Heteroskedasticity-robust standard errors are provided in parentheses. All models include the number of children, religious belief, whether any family member is a medic, doctor’s recommendation on self-paid vaccines, travel cost, father’s occupation, mother’s occupation, father’s age, and mother’s age.

The mediating analyses for varicella vaccines (presented in Table 4) and flu vaccines (listed in Table 5) display patterns similar to those observed for EV71 vaccines. For these three types of self-paid vaccines, income level emerges as the principal pathway through which education level influences vaccination willingness. Information and awareness do not significantly mediate this relationship. While trust level does serve as a potential pathway, its effect is relatively minor. Further examinations of the impact of a university degree on potential mediators are detailed in Supplementary material S2.

**Table 4:** Parental education and willingness to vaccinate with varicella vaccines.

Variables	Dependent variable: willingness to be vaccinated of varicella vaccination				
	Model 1	Model 2	Model 3	Model 4	Model 5
University degree	0.466** (0.198)	0.154 (0.203)	0.454** (0.198)	0.435*** (0.197)	0.132 (0.201)
Family income		0.514*** (0.079)			0.475*** (0.078)
Heard about it			0.241 (0.166)		0.168 (0.162)
Vital for children				0.576** (0.244)	0.550** (0.242)
Effective				1.011*** (0.250)	1.005*** (0.249)
Vital for the public				0.217 (0.199)	0.219 (0.200)
Worry about side effects				−0.492 (0.156)	−0.491*** (0.155)
Observations	1,994	1,979	1,993	1,970	1,954
Adjusted *R*^2^	0.042	0.062	0.042	0.080	0.098

*p < 0.01, **p < 0.05, ***p < 0.01. Heteroskedasticity-robust standard errors are provided in parentheses. All models include the number of children, religious belief, whether any family member is a medic, doctor’s recommendation on self-paid vaccines, travel cost, father’s occupation, mother’s occupation, father’s age, and mother’s age.

**Table 5:** Parental education and willingness to vaccinate with influenza vaccines.

Variables	Dependent variable: willingness to be vaccinated of varicella vaccination				
	Model 1	Model 2	Model 3	Model 4	Model 5
University degree	0.363* (0.202)	0.093 (0.208)	0.345* (0.203)	0.329* (0.198)	0.075 (0.205)
Family income		0.445** (0.082)			0.402*** (0.081)
Heard about it			0.152 (0.177)		0.0.28 (0.174)
Vital for children				0.717** (0.247)	0.693*** (0.248)
Effective				1.161*** (0.253)	1.172*** (0.254)
				0.210 (0.204)	0.204 (0.205)
Vital for the public				−0.599*** (0.159)	−0.605*** (0.159)
Worry about side effects	1,994	1,980	1,994	1,971	1,955
Observations	0.038	0.052	0.037	0.086	0.098

*p < 0.01, **p < 0.05, ***p < 0.01. Heteroskedasticity-robust standard errors are provided in parentheses. All models include the number of children, religious belief, whether any family member is a medic, doctor’s recommendation on self-paid vaccines, travel cost, father’s occupation, mother’s occupation, father’s age, and mother’s age.

Table 5 presents the regression analysis results with influenza vaccination intention as the dependent variable, comprising five models in total. Column (1) of Table 5 incorporates the “college education” variable and reports its effect on vaccination intention. Columns (2)–(4), respectively, incorporate the variables of “family income,” “information and cognitive factors,” and “vaccine confidence,” reporting their respective effects on vaccination willingness. Column (5) includes all the aforementioned variables as mediating factors and presents their collective influence, along with educational level, on vaccination willingness. The results show that the coefficient of a university degree is 0.363, which is statistically significantly positive at the 10% significance level, suggesting that parents with a college degree are more inclined to vaccinate their children against influenza. However, the adjusted *R*^2^ is only 0.038, indicating that the model has limited explanatory power. The coefficient for family income in column (2) is 0.445, which is statistically significantly positive at the 1% significance level, indicating that higher family income is associated with a greater willingness to receive vaccination. However, the coefficient associated with a college degree declined to 0.093 and became statistically insignificant, indicating that the influence of educational attainment on vaccination intention is entirely mediated by the variable of “family income.” This result supports Mechanism 1, suggesting that, ceteris paribus, family income exerts a mediating effect on the relationship between educational level and vaccination willingness. Column (3) demonstrates that the coefficient for information awareness is 0.152, yet the result is statistically insignificant. This indicates that informational and cognitive variables do not significantly influence vaccination intention. The coefficient for “Vaccines are important for children” provided in column (4) is 0.717, which is statistically significantly positive at the 1% significance level. Similarly, the coefficient for “Trust in vaccine effectiveness” is 1.161, also statistically significantly positive at the 1% significance level. These results indicate that trust in vaccines positively and reinforcingly influences vaccination willingness, such that higher levels of vaccine trust correspond to stronger vaccination intentions. The coefficient for “concern about side effects” is −0.599, which is statistically significant at the 1% significance level, indicating that concerns about vaccine side effects reduce vaccination willingness. The coefficient for “university education” declines to 0.329, showing positive significance at the 10% level. This suggests that vaccine trust partially mediates the relationship between education level and vaccination intention. In column (5), after the simultaneous inclusion of the three mediating variables, the coefficient for “family income” is 0.402, while the coefficient for “trust in vaccine effectiveness” is 1.172, which is significantly positive at the 1% significance level. Conversely, the coefficient for “concern about side effects” is −0.605, which is significantly negative at the 1% level. Meanwhile, the coefficient for “university education” decreases to 0.075 and becomes statistically insignificant. These results indicate that mediating variables such as family income and vaccine-related trust collectively exert a full mediating effect between educational attainment and vaccination willingness (Figure 1).

**Figure 1:**
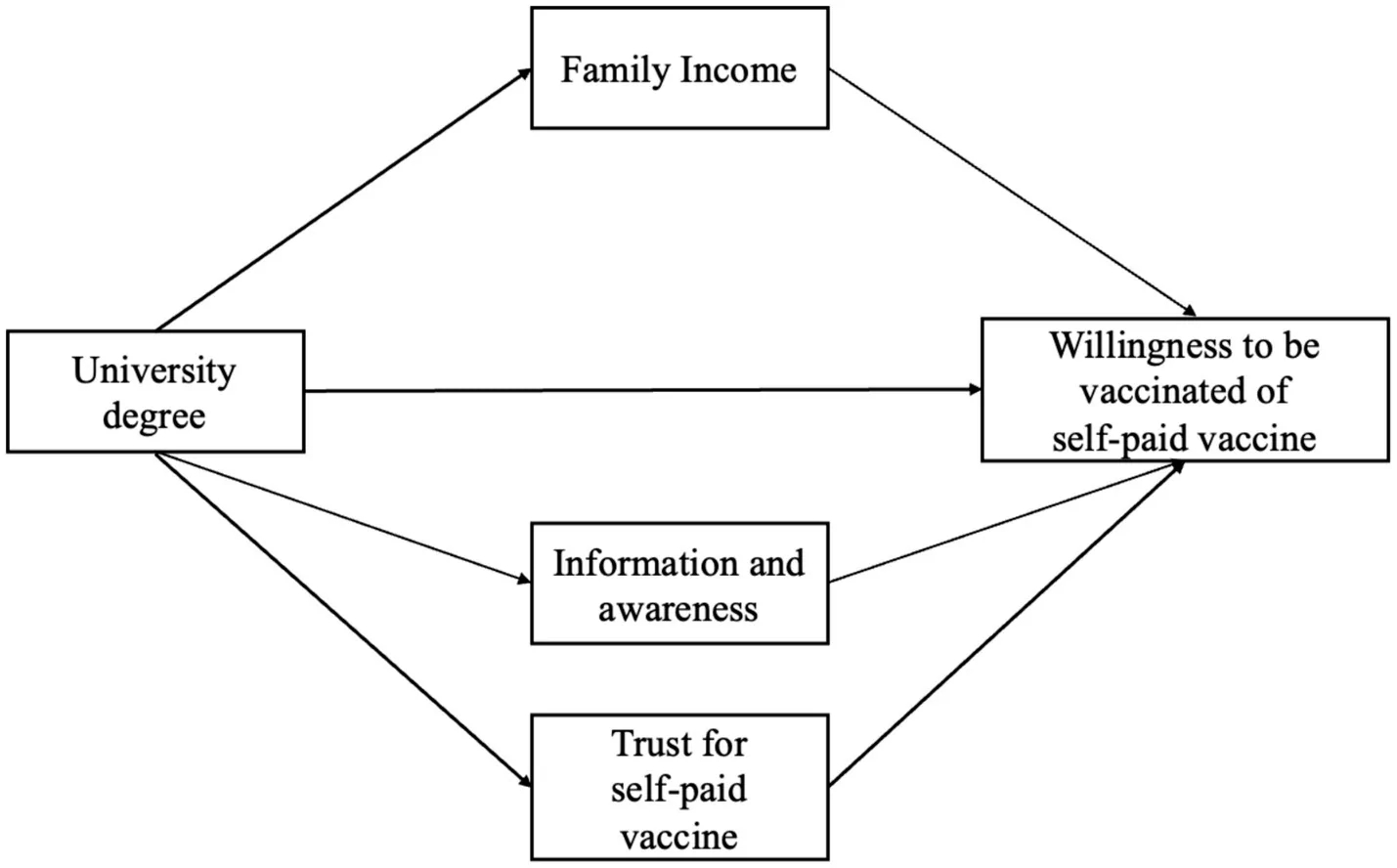
Potential mediating pathway.

## 4 Discussion

This study systematically verifies the mechanism of action of relevant variables such as family income, information and awareness, and trust in education level and vaccine willingness through regression analysis of four common self-paid vaccines: pentavalent vaccine (diphtheria, tetanus, acellular pertussis [whooping cough], Inactivated poliovirus [polio], and *haemophilus influenzae* type b [Hib] [DTaP-IPV-Hib]), EV71, varicella, and influenza in Figures 2–4. The findings reveal that two categories of variables—family income and trust in vaccines—mediate the relationship between education level and vaccination willingness. However, the proportion of the mediating effect attributable to family income differs across willingness to receive different types of vaccines. The mediating variable information and awareness exhibit differential effects within the mechanism through which educational attainment influences vaccination willingness. It demonstrates only partial mediation in the context of DTaP-IPV-Hib acceptance, whereas no significant mediating effect is observed for EV71, varicella, or influenza vaccines. To effectively enhance the willingness of self-paid children to receive vaccinations, o the one hand, it is suggested to improve the medical security policy for self-paid vaccination and to establish a support system that integrates medical, community, and educational resources to address economic barriers. For example, considering implementing reimbursement ratios for some self-paid vaccines (29, 30), providing appropriate rewards such as small subsidies to families who voluntarily self-paid vaccination and other related work, to alleviate family economic pressure and strengthen the defense line of family economic stability. On the other hand, it is recommended to optimize the multi-dimensional promotion and popularization layout, adopt hierarchical promotion and education based on the willingness to receive different vaccines through multiple channels and targeted measures, and pay special attention to the promotion and popularization of high cognitive threshold vaccines such as DTaP, IPV, Hib, and their awareness rate. For example, considering setting up temporary vaccination sites, implementing group appointments, strengthening communication strategies and trust counseling for pediatric and community nursing services (31), equipping mobile cold chain equipment and other related work, by improving the convenience of vaccination, parents’ awareness of vaccine characteristics and perceived benefits can be maximized, promoting the standardization and normalization of self-funded vaccination work, and further ensuring public health safety.

**Figure 2:**
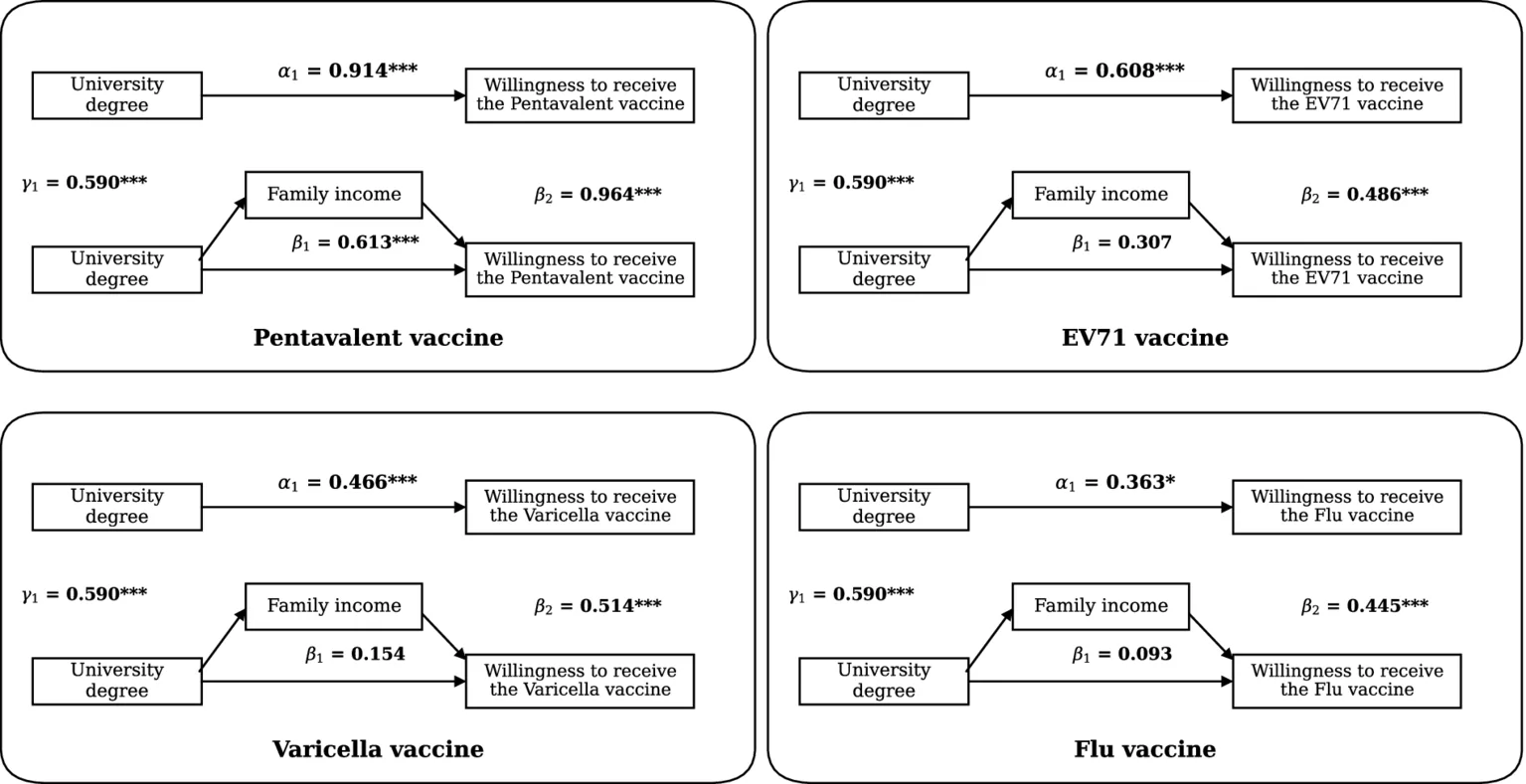
Mediating effect attributable to family income. *p < 0.01, **p < 0.05, ***p < 0.01. Supplementary material S1 shows different regression functions of α, β, and γ. α_1_ represents the total effect of a university degree on WTV, β_1_ represents the direct effect of a university degree on WTV, and γ_1_β_2_ represents the indirect effect of a university degree on WTV through family income.

**Figure 3:**
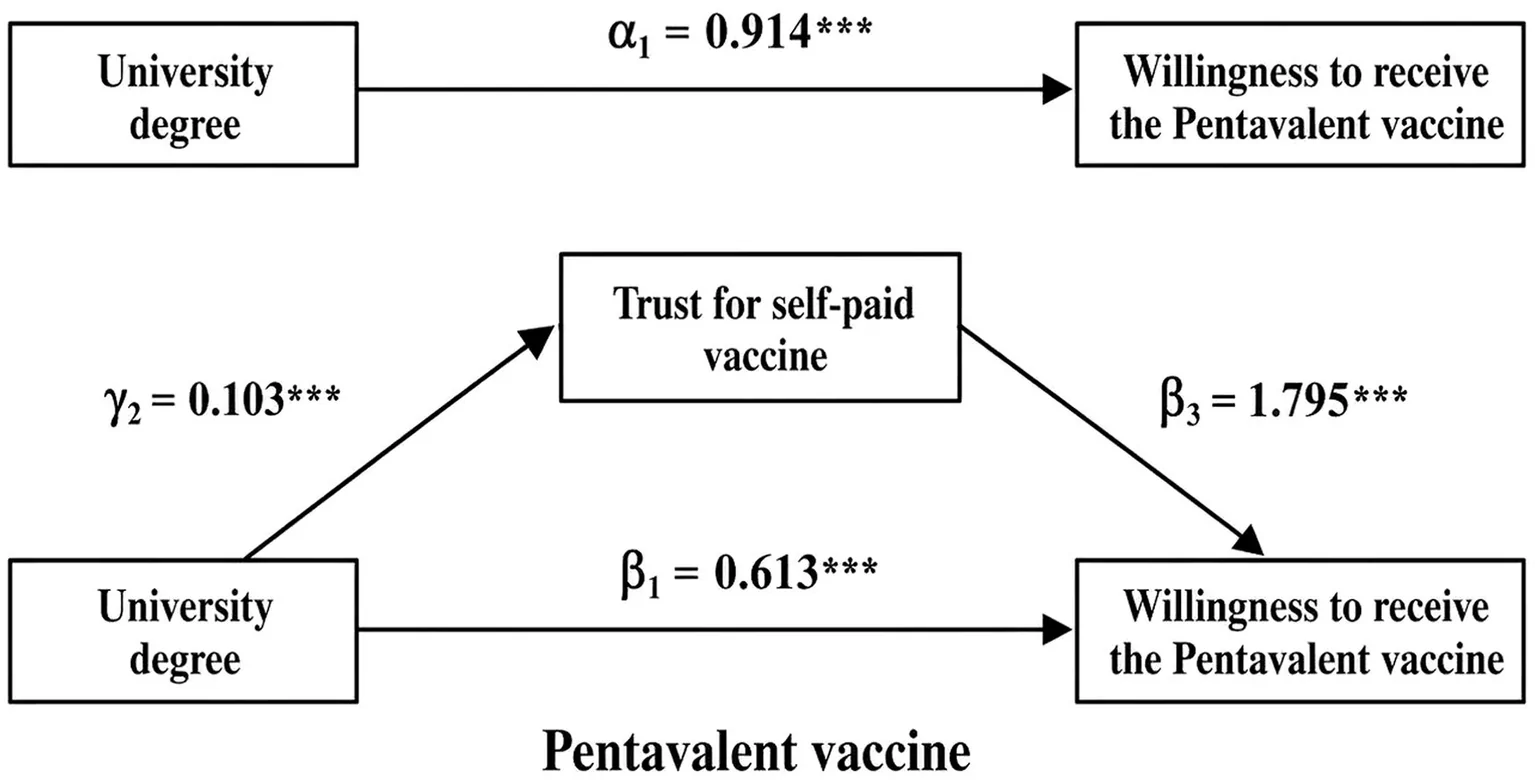
Mediating effect attributable to trust for self-paid vaccine. *p < 0.01, **p < 0.05, ***p < 0.01. Supplementary material S1 shows different regression functions of α, β, and γ. α_1_ represents the total effect of a university degree on WTV, β_1_ represents the direct effect of a university degree on WTV, and γ_2_β_3_ represents the indirect effect of a university degree on WTV through trust in the self-paid vaccine.

**Figure 4:**
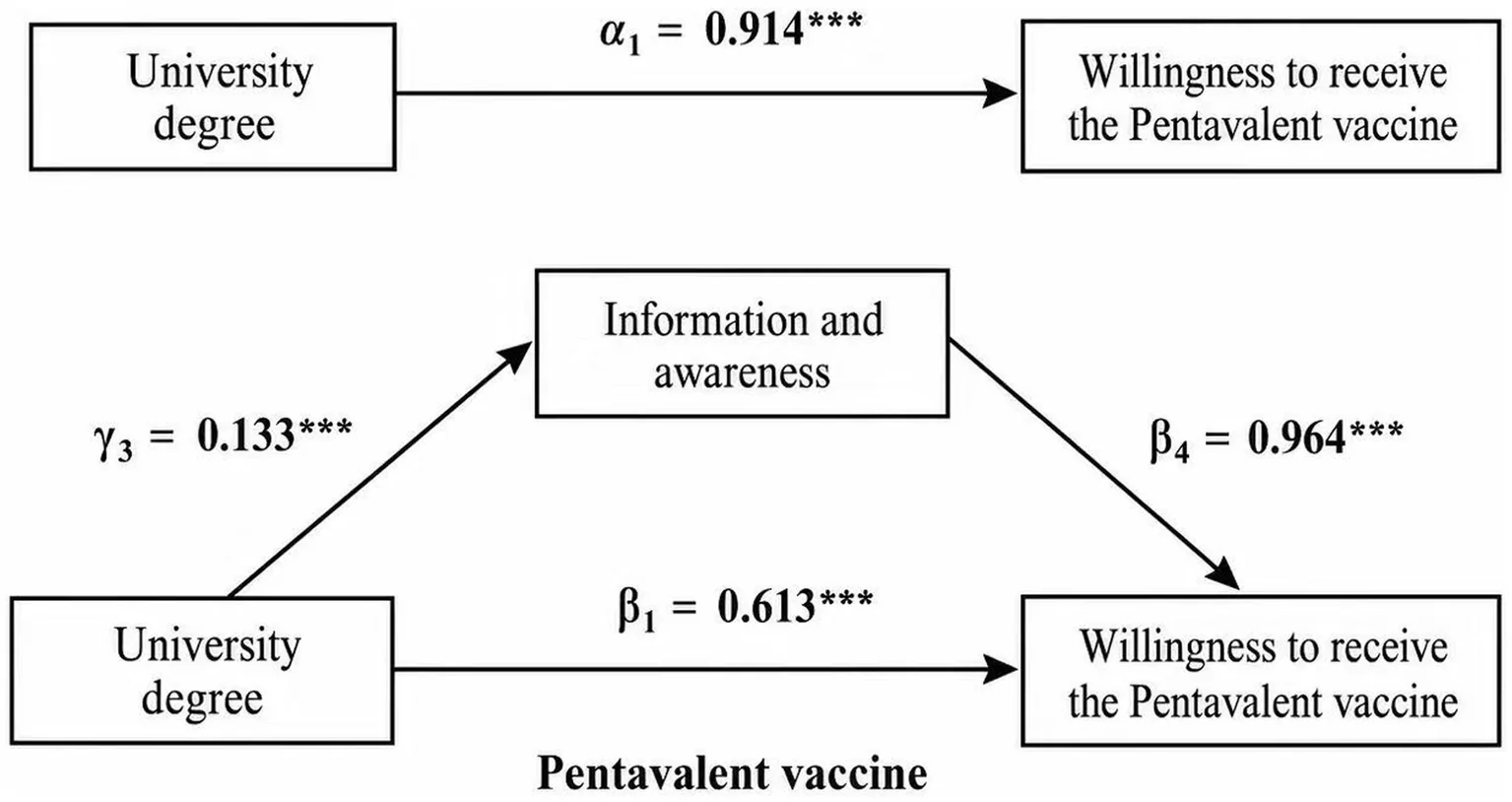
Mediating effect attributable to information and awareness. *p < 0.01, **p < 0.05, ***p < 0.01. Supplementary material S1 shows different regression functions of α, β, and γ. α_1_ represents the total effect of a university degree on WTV, β_1_ represents the direct effect of a university degree on WTV, and γ_3_β_4_ represents the indirect effect of a university degree on WTV through information and awareness.

### 4.1 Family income plays a significant positive role in promoting the influence of education level on willingness to vaccinate

Family income plays a significant positive role in the process by which education level influences the willingness to pay for self-paid vaccines. According to the results, for EV71, varicella, and influenza vaccines, after incorporating the family income variable, the coefficient of the college degree variable decreases and becomes statistically insignificant, while family income remains significantly positive. This suggests that education level does not directly affect vaccination willingness but is indirectly affected by improving family economic conditions. Family income fully mediates the effect of education level on vaccination willingness, whereas for the DTaP-IPV-Hib vaccine, it partially mediates this effect. To some extent, higher education levels correspond to better family economic conditions, which in turn lead to a stronger willingness to get vaccinated (21, 32), thus validating the first proposed mechanism (20). Moreover, even in economically prosperous Southern California, experiencing economic hardship was significantly associated with higher vaccine hesitancy among high-risk racial/ethnic minority communities, underscoring strong context-specific heterogeneity in the socioeconomic patterning of vaccine attitudes (20). Also, evidence is mixed: some studies report that higher socioeconomic status is associated with greater vaccine hesitancy, potentially due to greater exposure to diverse (including non-official) negative information and lower perceived infection risk, highlighting the role of information channels and trust differences (16, 19).

The proportion of the mediating effect of family income on the education effect varies significantly across different vaccine types. Among them, the mediating effects of the influenza and the varicella vaccines are the highest, reaching 72.3 and 65.1%, respectively, followed by the EV71 vaccine, while the DTaP-IPV-Hib vaccine shows a relatively lower effect. Variations across vaccine types suggest that financial factors may influence the willingness to use self-paid vaccines; however, this interpretation should be approached with caution. The larger indirect-effect estimates seen for some vaccines may mean that parents think more about their family’s finances when they think about vaccines that that reduce the out-of-pocket costs (26). These findings should not be viewed as proving that fewer financial barriers are useful, since this has already been seen before. However, our results provide context-specific results from China, suggesting that household financial resources may be one such reason for willingness to pay for self-payable vaccines. In this context, policy measures that reduce out-of-pocket costs, such as subsidies, reimbursements, or other forms of financial aid, may be of particular interest and should be considered alongside efforts to improve vaccine information and trust. In this context, policy measures that reduce out-of-pocket costs, such as lowering the prices of self-paid vaccines (33), increasing the government’s financial subsidies for self-paid vaccines (33), or including some self-paid vaccines in the medical insurance coverage.

### 4.2 Trust in vaccines is a mediating variable between education level and vaccination willingness

Trust in self-paid vaccines serves as a key mediating variable through which education level influences vaccination intention, thereby validating the third mechanism. The regression results for all vaccine types show that the coefficients for “importance to children” and “effectiveness” are significantly positive, while the coefficient for “worries about side effects” is significantly negative. This indicates that the more parents recognize the value of vaccines for children’s health and trust their protective effects, the stronger their willingness to vaccinate will be (32, 34–36). Trust in self-paid vaccines plays a critical mediating role in the influence of education level on vaccination willingness. Taking the DTaP-IPV-Hib vaccine as an example, in the model that does not fully incorporate trust variables (column 4), the coefficient for university education is 0.528 (*p* < 0.01). However, when all trust variables and control variables are included in column 5, the coefficient for university education drops to 0.212, with a significant decrease in significance and no statistical significance. This reflects that highly educated parents typically possess stronger abilities to acquire scientific information, enabling them to form positive judgments regarding the importance and effectiveness of self-paid vaccines, while also being able to more rationally assess vaccine side effects and reduce excessive concerns. Therefore, to improve vaccination rates for self-paid vaccines, it is necessary to enhance public trust in vaccination (34, 36). And efforts must focus on strengthening positive publicity and education on the importance, safety, and effectiveness of vaccination, and effectively controlling and supervising (37) exaggerated bad reports and untrue information sources (15, 34) on multiple platforms such as digital media.

### 4.3 Information and awareness do not play a significant mediating role between education level and vaccination willingness

Information and awareness variables partially mediated the effect of education level on willingness to receive the DTaP-IPV-Hib vaccine, but did not mediate the effect on willingness to receive other types of vaccines. According to the regression results for DTaP-IPV-Hib vaccine willingness (column 5 of Table 2), the coefficient for “Awareness of Vaccines” is 0.501, demonstrating a statistically significant positive effect at the 5% level, while the coefficient for “Understanding Differences Among Vaccines” is 0.625, indicating a highly significant positive influence at the 1% level. This indicates that higher parental awareness of the DTaP-IPV-Hib vaccine and a clearer understanding of its differences from single vaccines are associated with stronger vaccination willingness. However, in the regression results for EV71, varicella, and influenza vaccines, information and awareness variables do not influence vaccination willingness.

Information and awareness partially mediate the relationship between university education level and vaccination willingness for the DTaP-IPV-Hib vaccine, but not in EV71, varicella, or influenza vaccines. Taking the DTaP-IPV-Hib vaccine as an example, after incorporating information and awareness variables, the regression coefficient for university education level decreases from 0.613 to 0.536, suggesting that parents with higher education levels are better able to fully understand and retain vaccine-related information, thereby enhancing vaccination willingness. By comparison, the coefficients for the “university education” variable in the other three vaccine categories remain consistent and statistically insignificant across models, indicating that the information cognition variable does not mediate. This result indicates that the promotion and popularity of different vaccines, as well as their awareness rates, have influenced people’s willingness to receive vaccinations to some extent. These findings suggest that financial support alone may not be sufficient in all vaccine contexts. For vaccines with higher cognitive thresholds, willingness may also depend on parents’ understanding of vaccine characteristics and perceived benefits (38). In summary, the economic pressure that self-paid vaccines impose on individual families should not be underestimated, but the public’s awareness and effectiveness in vaccine inoculation need to be further emphasized and strengthened. Hence, focused communication and health education provided by primary healthcare institutions and reputable health platforms should be considered alongside strategies to address financial barriers. Particularly for vaccines with high cognitive thresholds, such as the DTaP-IPV-Hib vaccine, the promotion of their combined immunization advantages, safety, and long-term health benefits could be strengthened through community nursing services, maternal and child health systems, and authoritative health platforms (22, 23, 26).

## 5 Limitations

First, we sample from three districts in Hangzhou rather than a nationwide probability sample. While our sampling is meant to reduce the over-representation of parents who want to get just self-paid vaccines, selection bias cannot be neglected. For such a purpose, our results should be interpreted with a limited extent in view of generalizability. The future studies on broader coverage can also be considered. Although the questionnaire was developed based on previous literature, an existing vaccine hesitancy measure, and preliminary field testing, it was not a fully validated psychometric instrument. Therefore, the future studies should further examine its construct validity and applicability in other regions and populations.

## 6 Conclusion

This study reveals that family income may be one way to link parents’ education to their willingness to use self-paid vaccines in China. Household financial resources seemed more closely related to willingness in the mediation models than to some perception-based variables; however, these results should be interpreted cautiously given the observational nature of the study. Also, because the study was conducted in three districts of one city rather than a nationwide probability sample, it may be inappropriate to interpret the results in the same way. The study concludes that further studies with lower financial barriers may also help encourage greater willingness to self-fund a vaccine. Policy choices like targeted subsidies, increased reimbursement, or other financial supports would likely be considered in China, but studies with stronger designs could still be needed to verify these pathway relationships and determine whether greater willingness is a function of vaccine uptake. This study also suggests that family income may represent one potential pathway linking parental education with willingness to use self-paid vaccines in China. Among the factors examined, household financial resources appeared to be more strongly related to willingness in the mediation models than some perception-based variables. However, these findings should be interpreted cautiously, given the observational nature of the study. The results imply that, in addition to improving vaccine-related information and trust, reducing financial barriers may be important for increasing willingness to use self-paid vaccines. Policy approaches such as targeted subsidies, expanded reimbursement, or other financial support mechanisms may therefore deserve consideration in the Chinese context. The further studies using stronger designs are needed to confirm these pathway relationships and to assess whether increased willingness translates into actual vaccine uptake.

## Data Availability

The datasets used and/or analyzed during the current study are available from the corresponding author upon reasonable request.
