# Supplementary material for: Parental education and willingness to vaccinate children with self-paid vaccines in Hangzhou, China: a cross-sectional study
Source: Front Public Health. 2026 Oct 14;14:1804018. doi: 10.3389/fpubh.2026.1804018 (PMC13713807; doi:10.3389/fpubh.2026.1804018)
Supplement: Supplementary file 1 [file Supplementary_file_1.docx]

**Supplementary Materials**

**Supplementary Material S1: Regression Functions**

$${WTV}_{i}=\alpha_{0}+ {\alpha_{1}UniversityDegree}_{i}+\boldsymbol{control}_{\boldsymbol{i}}\boldsymbol{\cdot\zeta}+\varepsilon_{1}$$

$${WTV}_{i}=\beta_{0}+ {\beta_{1}UniversityDegree}_{i}+{{\beta_{2}Mediator}_{i}\boldsymbol{+contro}l}_{\boldsymbol{i}}\boldsymbol{\cdot\eta}+\varepsilon_{2}$$

$${Mediator}_{i}=\gamma_{0}+ {\gamma_{1}UniversityDegree}_{i}+\boldsymbol{control}_{\boldsymbol{i}}\boldsymbol{\cdot\varsigma}+\varepsilon_{3}$$

**Supplementary Material S2: The Impact of University Education on Mediators**

|  | Dependent variables | | | | |  |
| --- | --- | --- | --- | --- | --- | --- |
|  | (1) | (2) | (3) | (4) | (5) | |
|  | Family Income | Heard about it | Know the difference | Vital for children | Effective | |
| University Degree | $0.590$*** | $0.133$*** | 0.174*** | 0.006 | 0.026 | |
|  | $(0.057)$ | $(0.024)$ | (0.028) | (0.025) | $(0.025)$ | |
| Observations | $1,987$ | $2,001$ | $1,916$ | $1,993$ | $1,993$ | |
| Adjusted $R^{2}$ | 0.181 | 0.089 | 0.057 | 0.026 | 0.041 | |
|  |  |  |  |  |  | |
|  | (6) | (7) | (8) | (9) |  | |
|  | Vital for the public | Worry about side effects | Prefer Imported | Trust |  | |
| University Degree | $0.039$ | $0.048$* | 0.131*** | 0.103*** |  | |
|  | $(0.026)$ | $(0.028)$ | (0.027) | (0.025) |  | |
| Observations | $1,992$ | $1,987$ | $1,990$ | $2,002$ |  | |
| Adjusted $R^{2}$ | 0.028 | 0.006 | 0.043 | 0.057 |  | |

Table s1: Pentavalent vaccination

*Note: ** $p<0.1$*, *** $p<0.05$*, **** $p<0.01$*. Heteroskedasticity robust standard error is in parentheses. All models include the number of children, religious belief, whether any family member is medics, doctor’s recommendation on self-paid vaccines, travel cost, father’s occupation, mother’s occupation, father’s age, and mother’s age.*

|  | Dependent variables | | |
| --- | --- | --- | --- |
|  | (1) | (2) | (3) |
|  | Family Income | Heard about it | Vital for children |
| University Degree | $0.590$*** | $0.027$ | 0.006 |
|  | $(0.057)$ | $(0.024)$ | (0.025) |
| Observations | $1,987$ | $2,001$ | $1,993$ |
| Adjusted $R^{2}$ | 0.181 | 0.013 | 0.026 |
|  |  |  |  |
|  | (4) | (5) | (6) |
|  | Effective | Vital for the public | Worry about side effects |
| University Degree | 0.026 | $0.039$ | $0.048$* |
|  | $(0.025)$ | $(0.026)$ | $(0.028)$ |
| Observations | $1,993$ | $1,992$ | $1,987$ |
| Adjusted $R^{2}$ | 0.041 | 0.028 | 0.006 |

Table s2: EV71-vaccination

*Note: ** $p<0.1$*, *** $p<0.05$*, **** $p<0.01$*. Heteroskedasticity robust standard error is in parentheses. All models include the number of children, religious belief, whether any family member is medics, doctor’s recommendation on self-paid vaccines, travel cost, father’s occupation, mother’s occupation, father’s age, and mother’s age.*

|  | Dependent variables | | |
| --- | --- | --- | --- |
|  | (1) | (2) | (3) |
|  | Family Income | Heard about it | Vital for children |
| University Degree | $0.590$*** | $0.040$ | 0.006 |
|  | $(0.057)$ | $(0.027)$ | (0.025) |
| Observations | $1,987$ | $2,001$ | $1,993$ |
| Adjusted $R^{2}$ | 0.181 | 0.004 | 0.026 |
|  |  |  |  |
|  | (4) | (5) | (6) |
|  | Effective | Vital for the public | Worry about side effects |
| University Degree | 0.026 | $0.039$ | $0.048$* |
|  | $(0.025)$ | $(0.026)$ | $(0.028)$ |
| Observations | $1,993$ | $1,992$ | $1,987$ |
| Adjusted $R^{2}$ | 0.041 | 0.028 | 0.006 |

Table s3: Varicella vaccination

*Note: ** $p<0.1$*, *** $p<0.05$*, **** $p<0.01$*. Heteroskedasticity robust standard error is in parentheses. All models include the number of children, religious belief, whether any family member is medics, doctor’s recommendation on self-paid vaccines, travel cost, father’s occupation, mother’s occupation, father’s age, and mother’s age.*

|  | Dependent variables | | |
| --- | --- | --- | --- |
|  | (1) | (2) | (3) |
|  | Family Income | Heard about it | Vital for children |
| University Degree | $0.590$*** | $0.106$*** | 0.006 |
|  | $(0.057)$ | $(0.026)$ | (0.025) |
| Observations | $1,987$ | $2,001$ | $1,993$ |
| Adjusted $R^{2}$ | 0.181 | 0.037 | 0.026 |
|  |  |  |  |
|  | (4) | (5) | (6) |
|  | Effective | Vital for the public | Worry about side effects |
| University Degree | 0.026 | $0.039$ | $0.048$* |
|  | $(0.025)$ | $(0.026)$ | $(0.028)$ |
| Observations | $1,993$ | $1,992$ | $1,987$ |
| Adjusted $R^{2}$ | 0.041 | 0.028 | 0.006 |

Table s4: Flu vaccination

*Note: ** $p<0.1$*, *** $p<0.05$*, **** $p<0.01$*. Heteroskedasticity robust standard error is in parentheses. All models include the number of children, religious belief, whether any family member is medics, doctor’s recommendation on self-paid vaccines, travel cost, father’s occupation, mother’s occupation, father’s age, and mother’s age.*
